# VEGF vascularization pathway in human intervertebral disc does not change during the disc degeneration process

**DOI:** 10.1186/s13104-018-3441-3

**Published:** 2018-05-22

**Authors:** Simona Capossela, Alessandro Bertolo, Kapila Gunasekera, Tobias Pötzel, Martin Baur, Jivko V. Stoyanov

**Affiliations:** 1 Biomedical Laboratories, Swiss Paraplegic Research, 6207 Nottwil, Switzerland; 2 Swiss Paraplegic Centre, Nottwil, Switzerland; 3 Cantonal Hospital of Lucerne, Lucerne, Switzerland

**Keywords:** VEGFR1, FLT1, Vascularization, Intervertebral disc

## Abstract

**Objective:**

During degeneration of the intervertebral disc ingrowth of blood vessels and nerves into the disc are associated with back pain. Vascular endothelial growth factors promote vasculogenesis by binding to the membrane vascular endothelial growth factor receptor 1, while shorter soluble forms of this receptor can inhibit vascularization. We hypothesized that membrane and soluble receptor forms might change between stages of intervertebral disc degeneration.

**Results:**

Expression of soluble and membrane forms of vascular endothelial growth factor receptor 1 in human degenerated intervertebral discs and healthy bovine caudal discs was assessed by qRT-PCR and immunoblot. Comparative microarray meta-analysis across disc degeneration grades showed that membrane and soluble forms of this receptor, together with other components of classic vascularization pathways, are constitutively expressed across human disc degeneration stages. Contrary to our hypothesis, we observed that expression of the classic vascularization pathway is stable across degeneration stages and we assume that soluble vascular endothelial growth factor receptor 1 does not contribute to prevent disc degeneration. However, we observed increased expression levels of genes involved in alternative vascularization signalling pathways in severely degenerated discs, suggesting that abnormal vascularization is part of the pathological progression of disc degeneration.

**Electronic supplementary material:**

The online version of this article (10.1186/s13104-018-3441-3) contains supplementary material, which is available to authorized users.

## Introduction

The intervertebral disc (IVD) is a complex avascular structure composed of outer annulus fibrosus (AF) and central nucleus pulposus (NP), sandwiched by cartilage endplates [1]. Essential nutrients for cell activity are supplied by diffusion through the dense extracellular matrix, from the blood vessels at the endplates and the disc margins [2]. Aggrecan, the major proteoglycan in human IVD, has been demonstrated to inhibit nerve growth [3] and blood vessels migration into the disc [4]. Loss of proteoglycans and water content in degenerated disc tissues [5] is associated with disc vascularization and innervation [6]. Vascular and neural ingrowth into IVD has been correlated with the presence of vascular endothelial growth factors (VEGF) and their receptors [7]. VEGF regulate blood and lymphatic vessel development by binding to cell membrane receptors and activating specific signalling pathways [8]. VEGF receptor 1 (VEGFR1; also known as FLT1) is a transmembrane receptor for VEGFA, which promotes angiogenesis and vascular permeability. While VEGFR1 gene encodes the full-length membrane receptor (mVEGFR1), an alternative pre-mRNA splicing encodes for a negative regulator soluble receptor (sVEGFR1), which binds VEGFA and decreases its bio-availability [9]. sVEGFR1 has shorter and distinct C-terminus and lacks the transmembrane region [10]. In such way the expression of mVEGFR1 and sVEGFR1 can be utilised to regulate the local response to VEGF [11]. Interestingly, sVEGFR1 has an essential role in conferring corneal avascularity in diverse mammals [12] and reduced levels of this protein were found in people with age-related macular degeneration [13]. Recently, it has been shown that inhibitory factors, present in conditioned media generated from porcine notochordal-enriched NP, can inhibit in vitro angiogenesis by suppressing VEGF signalling pathway [14]. In this study we tested the hypothesis that human IVD expresses sVEGFR1 as an additional way to maintain its avascularity and that sVEGFR1 expression may change with disc degeneration.

## Main text

### Materials and methods

#### Isolation and culture of human and bovine disc cells

Human degenerated IVD fragments were obtained from patients undergoing surgical intervention (Additional file 1: Table S1), after informed consent and approval by ethics committee of North and Central Switzerland (EKNZ). Degeneration grade was determined by magnetic resonance imaging (MRI) and based on surgeons expertise, according to the Pfirrmann’s modification of the Thompson classification. Five-score grades from healthy (grade I) to the most advanced degenerated (grade V) disc are defined in terms of sequential changes to MRI features: disc height, distinction between AF and NP, brightness and uniformity of the NP [15].

Human degenerated IVD fragments were digested with 0.05% Collagenase Type-2 (Worthington—Bioconcept, Allschwil, Switzerland) in DMEM/F12 + GlutaMAX with 5% fetal bovine serum (FBS) (Gibco—Paisley, UK) for 6 h at 37 °C. As healthy human IVD (control) were inaccessible for ethical reason, we isolated discs from bovine tail obtained from a local slaughterhouse. Healthy bovine disc fragments were digested with 0.3% pronase (Sigma, Buchs, Switzerland) in DMEM/F12 + GlutaMAX with 5% FBS, for 1 h at 37 °C, then further digested with Collagenase overnight at 37 °C. Disc cells were expanded in monolayer culture in DMEM/F12 + GlutaMAX supplemented with 10% FBS (Gibco) and 5 ng/ml recombinant bFGF (Peprotech—LuBioScience, Lucerne, Switzerland) at 37 °C in a humid atmosphere containing 5% CO_2_.

#### RNA extraction and gene expression

IVD tissues stored at − 80 °C were disintegrated mechanically while still frozen and lysed in RNA Lysis Buffer (Aurum Total Mini Kit, Bio-Rad—Cressier, Switzerland). Disc cells in monolayer were trypsinized and the pellet was dissolved in RNA Lysis Buffer. Total RNA was extracted (Aurum Total Mini Kit, Bio-Rad) and used for synthesis of cDNA (SuperScript VILO cDNA Synthesis Kit, Invitrogen—LuBioScience). Template cDNA was mixed with PCR reaction solution (IQ SYBR Green Supermix, Bio-Rad) containing 0.25 μM specific primers (Additional file 2: Table S2). Quantitative PCR (qRT-PCR) reactions were carried out in duplicate in 96-well plates (Bio Rad) for 40 amplification cycles, followed by melting curve analysis. Relative quantification was calculated based on the 2^−ΔΔCt^ method and normalized to GAPDH.

#### Immunoblotting

Flow-through washes from the RNA extraction procedure were stored overnight at − 20 °C to allow protein precipitation, and then centrifuged at 10,000*g* for 15 min at 4 °C. Protein pellets were washed three times with 70% ethanol and resuspended in CelLytic M Buffer (Sigma) with Protease Inhibitor Cocktail (Sigma). In Western blot assays, proteins were separated by SDS-PAGE using Tris–Glycine 5–15% gradient gels (Bio-Rad) with a Protein Standard 10–250 kDa (Bio-Rad) and transferred onto a nitrocellulose membrane using the semi-dry Trans-Blot Turbo system (Bio-Rad). After blocking of non-specific sites with 5% milk (Rapilait, Migros, Switzerland) in PBS, membranes were incubated overnight at 4 °C with primary antibodies diluted in 5% milk: anti-VEGFR1 1:1000 (AF321 R&D System—Abingdon, UK); anti-soluble VEGFR1 1:50 (36–1100 Thermo Scientific—LuBioScience); anti β-Actin 1:10,000 (AC-15 Novus—LuBioScience). Secondary antibody HRP-conjugated (anti-mouse and anti-rabbit 1:10,000, anti-goat 1:20,000—Bethyl, LuBioScience) was incubated 1 h at room temperature in 5% milk-PBS and detected with chemiluminescence substrate (LumiGlo Reserve, KPL—Bio Concept, Allschwil, Switzerland). Acquisition was performed with digital SLR camera (Nikon D600—Nikon, Zürich, Switzerland) [16].

Western blot quantification was performed with ImageJ 1.49v.

#### Microarray data analysis

Two microarray data sets (GSE15227 and GSE23130) [17, 18] were downloaded from the Gene Expression Omnibus (GEO) using GEOquery, an R bioconductor package. These datasets have been generated from AF tissues of human degenerated IVD classified according to Thompson grading system. A quality check was performed using ArrayQualityMetrics to identify the arrays with poor quality. It was found that there were no notable deviations amongst 26 chips out of 38 chips. The intensities were re-calculated and normalized using Robust Multichip Average (RMA), Guanine Cytosine Robust Multi-Array (GCRMA) and Variance Stabilizing (VSN) methods. Having observed that the normalization could bring the intensity distribution of the selected 26 chips to similar characteristics, we decided to use these chips in our analysis (Additional file 3: Table S3). We then filtered out unwanted information (e.g. genes without entrez information, duplicated entrez gene identifiers etc.) together with genes having low variance, which would not pass the statistical tests for differential expression. Subsequently the filtered data sets were processed using limma R package to identify deferentially expressed genes. The results presented are based on VSN normalization.

#### Statistical analysis

For statistical analysis, we used non-parametric Mann–Whitney–Wilcoxon U test for independent variables. Data analysis was performed with SPSS version 24.0 for Windows (SPSS Inc.). Significance was indicated as *p < 0.05. After publication the data will be shared on Research Gate.

### Results

#### Human degenerated IVD express membrane and soluble VEGFR1

**Fig. 1:**
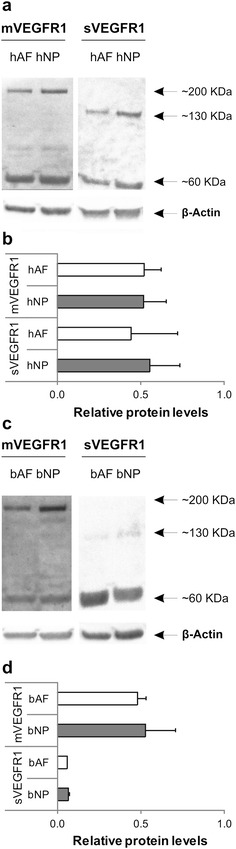
VEGFR1 isoforms in human degenerated and bovine healthy discs. A representative immunoblotting assay showed the expression of the full-length membrane form (~ 200 kDa), the soluble form (~ 130 kDa) and a cytoplasmic fragment (~ 60 kDa) in human degenerated (**a**) and healthy bovine (**c**) disc cells. β-Actin was used as control. Western blot quantification of membrane and soluble VEGFR1 was performed on human (**b**; n = 3) and bovine (**d**; n = 2) disc cells. Results represent an average of area (square pixels) ± SD normalized on βActin. White bars represent AF, grey bars NP. No statistical significance with non-parametric Mann–Whitney–Wilcoxon U test for independent variables

Immunoblotting assay with anti-VEGFR1 antibody against the N-terminus region of the protein showed the expression of the full-length membrane form (mVEGFR1 ~ 200 kDa) in human degenerated (Fig. 1a) and healthy bovine (Fig. 1c) disc cells. An antibody against the C-terminal region of VEGFR1 soluble form showed a band of ~ 130 kDa (sVEGFR1) in human degenerated (Fig. 1a) and healthy bovine (Fig. 1c) disc cells. Both antibodies showed a band at ~ 60 kDa. β-Actin was used as a control. Western blot quantification showed no significant differences between mVEGFR1 and sVEGFR1 in human degenerated IVD (n = 3—Fig. 1b) and healthy bovine discs (n = 2—Fig. 1d). Relative protein levels represent the average of the area (square pixels) normalized on β-Actin. qRT-PCR analysis with specific primers (Additional file 2: Table S2) designed in the unique regions of VEGFR1 gene to discriminate between different isoforms, showed that mVEGFR1 and sVEGFR1 were comparably expressed in human degenerated IVD tissues (n = 11) and disc cells cultures (n = 11) (Additional file 4: Figure S1).

#### Unchanged VEGF pathways and abnormal vascularization in IVD degeneration process

**Fig. 2:**
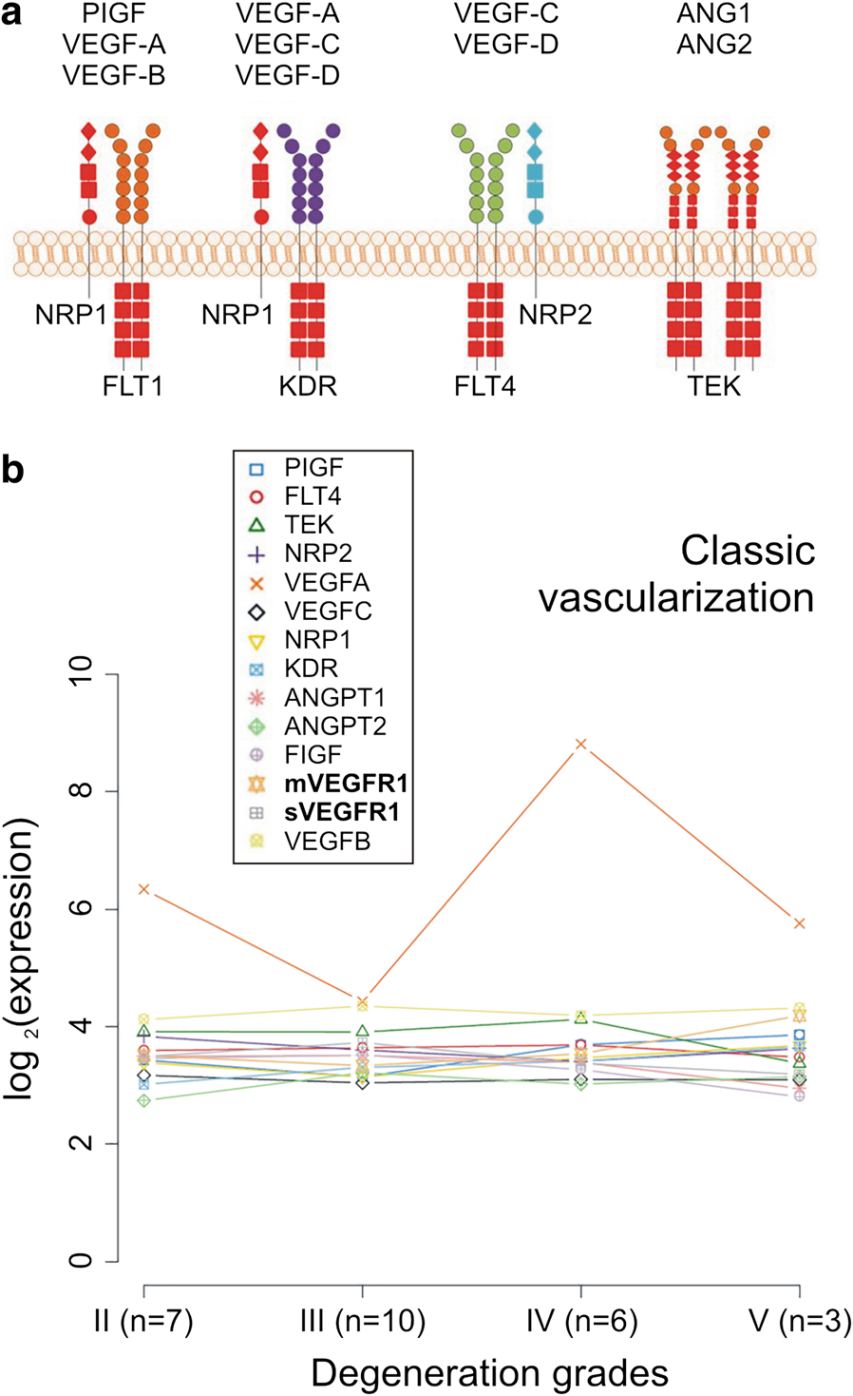
Unchanged VEGF pathways in IVD. **a** Schematic representation of members of the classic VEGF vascularization signalling pathway. **b** Microarray meta-analysis of 14 expression profiles remained unchanged through the degeneration grades (n = 26), except for vascular endothelial growth factor A (VEGFA). Axes of x indicate state of degeneration according to Thompson grading system, axes of y indicate average expression levels in log2. Results are based on VSN normalization

Comparative analysis of AF tissues (n = 26) expression profiles from two microarray data sets [17, 18] showed that sVEGFR1 and mVEGFR1, along with other members of the classic signalling vascularization pathway [19] (Fig. 2a), were unchanged at the level of transcript abundance through the degeneration grades (Fig. 2b). An exception was VEGFA, which exhibited a fluctuating expression between degeneration grades (Fig. 2b).

**Fig. 3:**
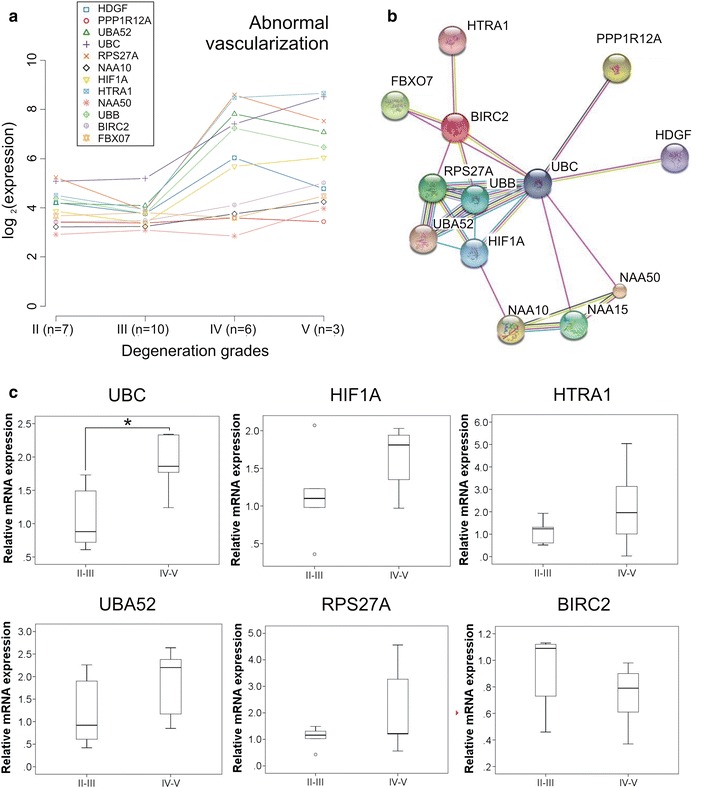
Abnormal vascularization in IVD. **a** Genes regulating abnormal alternative vascularization and other components of the interactome of hypoxia-inducible factor-1A (HIF1A) and high-temperature requirement A serine peptidase 1 (HTRA1) showed increased expression levels through degeneration grades (n = 26). Axes of x indicate state of degeneration, axes of y indicate average expression levels in log2. Results are based on VSN normalization. **b** Interactome of HIF1A and HTRA1 proposed by STRING database. **c** qRT-PCR validation of six selected genes regulating abnormal vascularization in degenerated grade II and III (n = 5), compared to severe degenerated grade IV and V (n = 5) AF tissues. Box plots represent relative mRNA expression normalized on GAPDH. The line across the box indicates the median, bubbles indicate outliers. Non-parametric Mann–Whitney–Wilcoxon U test for independent variables was used. Significance was showed as *p < 0.05

On the other hand, the expression levels of genes regulating abnormal alternative vascularization, such as hypoxia-inducible factor-1A (HIF1A) and High-Temperature Requirement A Serine Peptidase 1 (HTRA1) were significantly increased in degenerated AF grade IV and V (Fig. 3a). There were also increased expression levels of some of the components of the interactome of HIF1A and HTRA1 proposed by STRING database (Fig. 3b). Meta-analysis gene expression data are given in Additional file 5: Table S4 and Additional file 6: Table S5. The qRT-PCR validation of six selected genes regulating abnormal vascularization (Fig. 3c) showed significant increased expression levels of Ubiquitin C (UBC) in severe degenerated AF tissues (grade IV and V; n = 5), compared to grade II and III (n = 5). HIF1A, HTRA1, 40S Ribosomal Protein S27A (RPS27A) and Ubiquitin A-52 (UBA52) showed higher expression levels in degeneration grade IV and V, but the results were not significant.

### Discussion

In this study, we observed that gene expression in the classic vascular endothelial growth factor (VEGF) vascularization pathway is preserved across human intervertebral disc (IVD) degeneration stages and we propose that alternative vascularization pathways may be involved with the pathological progression of disc degeneration.

The degeneration of IVD is associated with vascularization and innervation [6]. VEGF promotes vasculogenesis binding to the full-length transmembrane vascular endothelial growth factor receptor 1 (mVEGFR1), while shorter soluble forms of this receptor (sVEGFR1) behave as competitive inhibitors of vascularization. Since sVEGFR1 has the essential role to preserve corneal avascularity in diverse mammals [12], we hypothesized that human IVD expresses the decoy sVEGFR1 as an additional way to maintain its avascularity and the expression of sVEGFR1 and mVEGFR1 may change during degeneration process. We expected that healthy avascular discs prevalently express the inhibitor soluble form, while the vascularized degenerated discs express more the membrane form. Contrary to our expectations, we showed that both forms were similarly expressed in human degenerated IVD tissues and disc cell cultures. By immunoblot, we observed the expression of mVEGFR1 (~ 200 kDa) and sVEGFR1 (~ 130 kDa), both isoforms well described in literature [10, 20–22]. It has been demonstrated that VEGFR1 can undergo proteolytic fragmentation, resulting in the formation of soluble form and cytoplasmic fragment of ~ 60 kDa [23]. We observed, by microarray meta-analysis, unchanged expression levels of sVEGFR1 and mVEGFR1 through the IVD degeneration grades, as well as of the other components of classic vascularization pathways [19], except for the fluctuating transcript levels expressed by VEGFA. VEGFR1 together with VEGFA are the imperative upstream components in a highly studied signalling pathway that regulates angiogenesis [19, 24–26]. The mechanism of neovascularization mediated by VEGF and its receptors has been closely correlated with inflammation, chronic back pain and accelerated IVD degeneration [27–32]. However, our meta-analysis rejected the initial hypothesis and showed that the classic vascularization signalling pathway is constitutively expressed across disc degeneration stages.

This leaves the option that other vascularization pathways may be involved in the pathological vascularization progression during IVD degeneration. Interestingly, the meta-analysis and our qRT-PCR validation experiments revealed number of genes-either known or proposed to regulate abnormal vascularization—with increased expression levels in severely degenerated discs. Above all, hypoxia-inducible factor-1A (HIF1A), which has been shown to regulate VEGF [33, 34] and induce angiogenesis [35], High-Temperature Requirement A Serine Peptidase 1 (HTRA1), which has a potential role in IVD degeneration [36, 37] and abnormal vascularization [38, 39], and Ubiquitin C (UBC), which was hypothesized to have a crucial role in inhibiting cell proliferation of annulus fibrosus in IVD degeneration [40].

In conclusion, this study showed that the classic VEGF vascularization pathway is unchanged across disc degeneration, advancing that decoy sVEGFR1 does not have a major role in protecting from disc degeneration process.

## Limitations

Our results allow us to speculate that IVD cells response to the degenerative processes may trigger an alternative vascularization signalling pathway activated by some of the components of the interactome of HIF1A and HTRA1. However, this is a new hypothesis, which needs to be tested.

In this study we used as healthy control caudal bovine discs, because healthy human IVD were inaccessible for ethical reason. Bovine discs have been proposed as a suitable biological and biomechanical model for studying human disc disorders, since they are very similar to the human IVD in terms of cell distribution, cell phenotype, disc composition, disc size and mechanical loading [41].

## Additional files

**Additional file 1: Table S1.** Demographic details of Intervertebral disc donors.

**Additional file 2: Table S2.** Human primers used in qRT-PCR.

**Additional file 3: Table S3.** Microarray samples information.

**Additional file 4: Figure S1.** (a) Schematic representation of transcript isoforms of VEGFR1. (UniProtKB-P17948). In red are marked identical N-terminus coding sequences, while in yellow the C-terminus unique sequences belonging to splicing isoforms. Specific primers (depicted in dotted lines) were designed in the unique regions to discriminate the different isoforms by qRT-PCR. (b) Agarose gel shows PCR products of VEGFR1 isoforms analysed in HUVEC; GAPDH was used as housekeeping gene. PCR product sizes were expressed in base pair (bp). (c) In human degenerated IVD tissues (n = 11) and (d) monolayer disc cell cultures (n = 11) mVEGFR1 and sVEGFR1 were similarly expressed. Soluble variants isoform 3 and isoform 4 were not detected by qRT-PCR. Box plots represent relative mRNA expression normalized on GAPDH in annulus fibrosus (AF) and nucleus pulposus (NP). The line across the box indicates the median, bubbles indicate outliers, stars indicate extreme values. No statistical significance with Non-parametric Mann–Whitney–Wilcoxon U test for independent variables.

**Additional file 5: Table S4.** Expression profiles of genes of classic angiogenesis signalling pathway.

**Additional file 6: Table S5.** Expression profiles of genes of abnormal angiogenesis signalling pathway.

## Abbreviations

IVD: intervertebral disc
mVEGFR1: membrane vascular endothelial growth factor receptor 1
sVEGFR1: soluble vascular endothelial growth factor receptor 1
AF: annulus fibrosus
NP: nucleus pulposus
VEGF: vascular endothelial growth factors
VEGFA: vascular endothelial growth factor A
HIF1A: hypoxia-inducible factor-1A
HTRA1: High-Temperature Requirement A Serine Peptidase 1
UBC: ubiquitin C
RPS27A: 40S Ribosomal Protein S27A
UBA52: ubiquitin A-52
BIRC2: baculoviral IAP repeat-containing protein 2
