# Supplementary figures and images for: VEGF vascularization pathway in human intervertebral disc does not change during the disc degeneration process
Source: BMC Res Notes. 2018 May 22;11:333. doi: 10.1186/s13104-018-3441-3 (PMC5963106; doi:10.1186/s13104-018-3441-3)

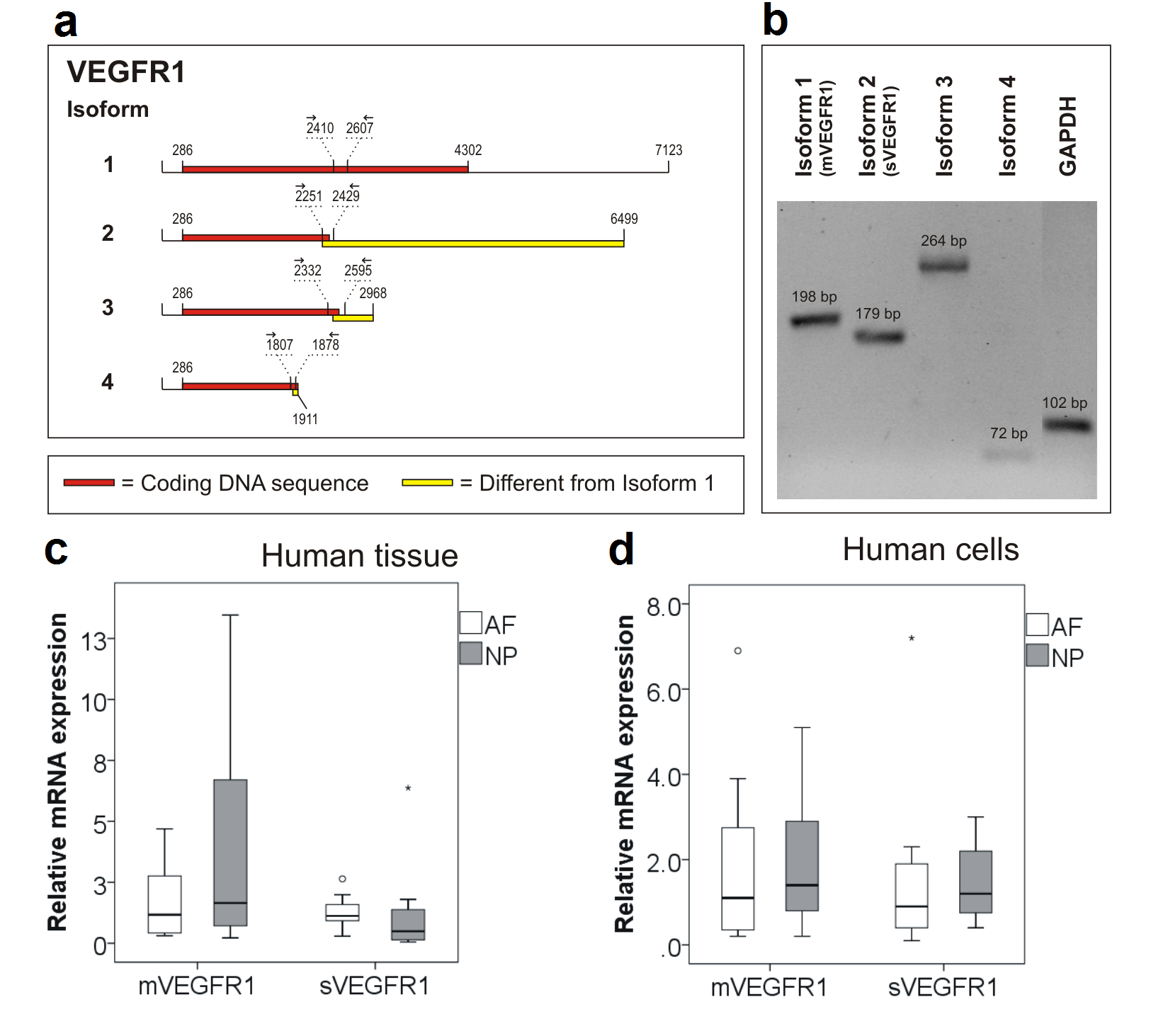

Supplement: Supplementary file 4 — Additional file 4: Figure S1. (a) Schematic representation of transcript isoforms of VEGFR1. (UniProtKB-P17948). In red are marked identical N-terminus coding sequences, while in yellow the C-terminus unique sequences belonging to splicing isoforms. Specific primers (depicted in dotted lines) were designed in the unique regions to discriminate the different isoforms by qRT-PCR. (b) Agarose gel shows PCR products of VEGFR1 isoforms analysed in HUVEC; GAPDH was used as housekeeping gene. PCR product sizes were expressed in base pair (bp). (c) In human degenerated IVD tissues (n = 11) and (d) monolayer disc cell cultures (n = 11) mVEGFR1 and sVEGFR1 were similarly expressed. Soluble variants isoform 3 and isoform 4 were not detected by qRT-PCR. Box plots represent relative mRNA expression normalized on GAPDH in annulus fibrosus (AF) and nucleus pulposus (NP). The line across the box indicates the median, bubbles indicate outliers, stars indicate extreme values. No statistical significance with Non-parametric Mann–Whitney–Wilcoxon U test for independent variables. [file 13104_2018_3441_MOESM4_ESM.tif]
